# Predictors of hepatocellular carcinoma recurrence associated with the use of direct‐acting antiviral agent therapy for hepatitis C virus after curative treatment: A prospective multicenter cohort study

**DOI:** 10.1002/cam4.2061

**Published:** 2019-03-22

**Authors:** Masahito Nakano, Hironori Koga, Tatsuya Ide, Ryoko Kuromatsu, Satoru Hashimoto, Hiroshi Yatsuhashi, Masataka Seike, Nobito Higuchi, Makoto Nakamuta, Satoshi Shakado, Shotaro Sakisaka, Satoshi Miuma, Kazuhiko Nakao, Yoko Yoshimaru, Yutaka Sasaki, Satoshi Oeda, Yuichiro Eguchi, Yuichi Honma, Masaru Harada, Kenji Nagata, Seiichi Mawatari, Akio Ido, Tatsuji Maeshiro, Shuichi Matsumoto, Yuko Takami, Tetsuo Sohda, Takuji Torimura

**Affiliations:** ^1^ Division of Gastroenterology, Department of Medicine Kurume University School of Medicine Fukuoka Japan; ^2^ Clinical Research Center National Hospital Organization Nagasaki Medical Center Nagasaki Japan; ^3^ Department of Gastroenterology, Faculty of Medicine Oita University Oita Japan; ^4^ Division of Gastroenterology National Kyusyu Medical Center Hospital Fukuoka Japan; ^5^ Department of Gastroenterology and Medicine Fukuoka University Faculty of Medicine Fukuoka Japan; ^6^ Department of Gastroenterology and Hepatology Nagasaki University Hospital Nagasaki Japan; ^7^ Department of Gastroenterology and Hepatology, Graduate School of Medical Sciences Kumamoto University Kumamoto Japan; ^8^ Liver Center Saga University Hospital Saga Japan; ^9^ Third Department of Internal Medicine University of Occupational and Environmental Health Kitakyushu Japan; ^10^ Department of Liver Disease University of Miyazaki Hospital Miyazaki Japan; ^11^ Digestive and Lifestyle Diseases Kagoshima University Graduate School of Medical and Dental Sciences Kagoshima Japan; ^12^ First Department of Internal Medicine, Faculty of Medicine University of the Ryukyus Okinawa Japan; ^13^ Fukuoka Tokushukai Medical Center Fukuoka Japan; ^14^ Department of Hepato‐Biliary‐Pancreatic Surgery and Clinical Research Institute National Kyushu Medical Center Hospital Fukuoka Japan; ^15^ Hepatology Division Japanese Red Cross Fukuoka Hospital Fukuoka Japan

**Keywords:** DAA, HCV, hepatocarcinogenesis, liver cancer, SVR

## Abstract

**Background:**

Previous studies have suggested an association between the use of direct‐acting antiviral agents (DAAs) for treating hepatitis C virus (HCV) infection and the resulting decrease in the incidence of hepatocellular carcinoma (HCC); however, it is unclear whether DAAs prevent the recurrence of HCC after curative treatment for HCC. This study aimed to prospectively investigate HCC recurrence and its predictors after curative treatment for HCC.

**Methods:**

A total of 3012 patients with chronic HCV infection, with or without cirrhosis, who were treated with DAAs were enrolled between January 1, 2015 and January 31, 2017 as per the institutional review board approved study protocol at 15 institutions, including 10 university hospitals and five high‐volume centers in the Kyusyu area of Japan. Of the 3012 patients, 459 patients who had HCC but were cured with surgery or ablation therapy (curative treatment) before the use of DAAs were included in the analysis.

**Results:**

During a mean follow‐up period of 29.4 months, 217 (47.2%) patients developed HCC recurrence. The median time to recurrence was 34.0 months, and the 1‐, 2‐, and 3‐year cumulative HCC recurrence rates were 27.1%, 43.4%, and 50.8%, respectively. The risk factors for HCC recurrence were the α‐fetoprotein (AFP) level before DAA therapy (*P* = 0.0047) and the number of curative treatments for HCC before DAA therapy (*P* < 0.0001).

**Conclusions:**

A high AFP level and multiple occurrences of HCC before DAA therapy are associated with a high risk for HCC recurrence after curative treatment. Follow‐up after DAA therapy should include special attention to the abovementioned risk factors.

## 1 INTRODUCTION

Chronic hepatitis C virus (HCV) infection is a major cause of liver cirrhosis and hepatocellular carcinoma (HCC).^1^, ^2^ HCC is a frequent consequence of HCV‐related cirrhosis, with an annual incidence of 1%‐8%.^3^ In addition, HCV infection may promote carcinogenesis; hence, its eradication will directly decrease the risk of developing HCC.^4^, ^5^ With the recent development of interferon (IFN)‐free direct‐acting antiviral agents (DAAs), high rates of sustained virological response (SVR) have been achieved in patients with chronic HCV infection.^6^, ^7^, ^8^

For many decades, IFN‐based regimens had been the standard care for treating HCV infection and reducing the risk of HCC development.^9^, ^10^, ^11^ Risk factors for HCC are old age, advanced liver fibrosis, male sex, post‐IFN α‐fetoprotein (AFP) levels, glucose metabolism disorders, lipid metabolism disorders, and alcohol intake.^12^

Although the SVR with DAAs reduces the incidence of HCC, an increase in unexpected early occurrence or recurrence of HCC after HCV elimination has been reported with DAA therapies.^13^, ^14^, ^15^, ^16^, ^17^ Recent studies have reported that HCV‐infected patients with HCC who had an initial complete response to hepatic resection or local ablation and subsequently had DAA‐related SVR experienced an increased risk of HCC recurrence.^16^, ^17^ The annual incidence of de novo HCC after inducing SVR with DAAs has been variably reported to be 3%‐5%, which is higher than that observed with IFN‐based therapy.^18^, ^19^, ^20^

Although previous studies have suggested an association between the use of DAAs for treating HCV infection and resulting decrease in the incidence of HCC, it is unclear whether DAAs used for treating HCV infection can prevent the recurrence of HCC after curative treatment for HCC. This study aimed to prospectively investigate the HCC recurrence and predictors of HCC recurrence after curative treatment for HCC.

## 2 PATIENTS AND METHODS

### 2.1 Patients

**Figure 1:**
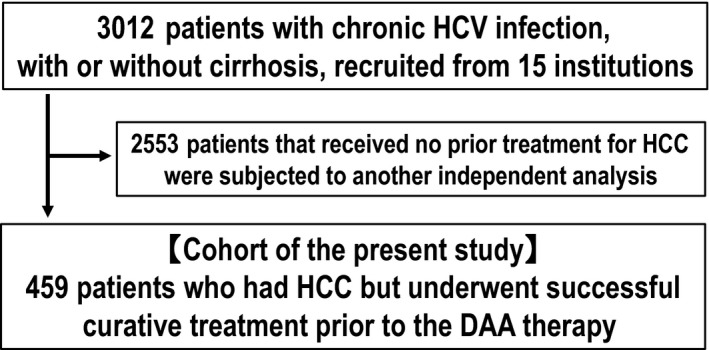
Flow chart detailing the criteria of patient selection

In this cohort study, we recruited 3012 patients with chronic HCV infection, with or without cirrhosis, who were treated with DAAs between January 1, 2015 and January 31, 2017. Of the 3012 patients, 459 patients who had HCC but were cured with surgery or ablation therapy (curative treatment) prior to the DAA therapy were included in the analysis. We specifically focused on the identification of predictors for HCC recurrence after DAA therapy in the 459 patients with precured HCC, while the remaining 2553 patients were subjected to an independent analysis (Figure 1), because the characteristics of the two populations, and thus the risk factors for HCC development were shown to be significantly different in previous studies.^11^ The SVR was confirmed by the absence of serum HCV‐RNA at 12 weeks after the discontinuation of DAAs. After the SVR was obtained, the patients were followed‐up prospectively. Patients were excluded if they had hepatitis B virus surface antigen or other forms of liver disease.

### 2.2 DAA therapy

The DAA therapy was provided at 15 institutions, including 10 universities and five high‐volume centers in the Kyusyu area of Japan. The therapeutic regimen included asunaprevir plus daclatasvir for 24 weeks, sofosbuvir (SOF)/ledipasvir for 12 weeks, SOF plus ribavirin for 12 weeks, and paritaprevir/ombitasvir/ritonavir for 12 weeks. The main outcome was HCC recurrence. The endpoint was the date on which HCC was diagnosed in the patients who had developed HCC or the date on which the absence of HCC was confirmed at the last follow‐up on January 31, 2018. The period of observation was defined as time from the initiation of DAA therapy to the endpoint.

### 2.3 Laboratory results

The results of routine tests for blood cell count, liver biochemical parameters, and AFP levels before starting the DAA therapy were collected. Aspartate aminotransferase to platelet count ratio index (APRI) was calculated as AST (/ULN)/platelet count [10^9^/L] × 100.^21^ Fibrosis‐4 (FIB‐4) index was calculated as age × AST/platelet count [10^9^/L] × ALT^1/2^.^22^ If patients had idiopathic thrombocytopenic purpura or other forms of hematological disorder, platelet count, APRI, and FIB‐4 index data were excluded. All patients had chronic HCV genotype (serotype) 1 or 2 infection. The attending physician clinically diagnosed the presence of cirrhosis. Alcoholism was defined as an average daily consumption of >75 g of ethanol. Patients with a bright liver, deep attenuation, and hepatorenal contrast on abdominal ultrasonography were diagnosed with fatty liver. A diagnosis of diabetes mellitus was made in accordance with the diagnostic criteria established by the Japan Diabetes Society.^23^ The protocol was approved by the institutional review boards of all hospitals, and the study was conducted in compliance with the principles of the Declaration of Helsinki. Written informed consent was obtained from all participating patients.

### 2.4 HCC diagnosis

HCC was confirmed either histologically or diagnosed using noninvasive criteria according to the European Association for the Study of the Liver.^24^ Intrahepatic lesions and vascular invasion were diagnosed using a combination of contrast‐enhanced computed tomography, magnetic resonance imaging, and ultrasonography.

### 2.5 Registration of the study

This study has been registered in the UMIN Clinical Trials Registry (http://www.umin.ac.jp/ctr/) (Trial ID: UMIN000027988).

### 2.6 Statistical analysis

The Cox proportional‐hazards regression model was used to estimate hazards ratios for risk factors. The cumulative incidence curve was determined using the Kaplan‐Meier method, and differences between groups were assessed using the log‐rank test. For all methods, the level of significance was set at *P* < 0.05. All statistical analyses were performed using the JMP pro 13 software (SAS Institute, Cary, NC).

## 3 RESULTS

### 3.1 Patient characteristics

Table 1 shows the characteristics of the enrolled patients (n = 459). Table 2 shows the characteristics of patients with or without HCC recurrence after the DAA therapy. There were significant differences in sex, platelet count, AFP level, the presence of cirrhosis, and number of curative treatments for HCC before the DAA therapy between the patients with and without HCC recurrence after the DAA therapy. Mean follow‐up period was 29.4 ± 6.8 months.

**Table 1:** Patient characteristics

	Patients (n = 459)
Age before DAA therapy (y)	74.9 ± 7.6
Sex (male/female)	269/190
AST level before DAA therapy (IU/L)	58.6 ± 31.1
ALT level before DAA therapy (IU/L)	48.5 ± 32.2
GGTP level before DAA therapy (IU/L)	48.8 ± 54.8
Platelet count before DAA therapy (×10^4^/μL)	10.7 ± 4.9
AFP level before DAA therapy (ng/mL)	28.5 ± 67.3
APRI before DAA therapy	2.32 ± 1.81
FIB‐4 index before DAA therapy	7.10 ± 4.16
Geno/serotype (1/2/1+2/unknown)	407/49/1/2
Habitual alcohol intake (presence/absence/unknown)	55/333/71
Diabetes mellitus (presence/absence/unknown)	128/315/16
Fatty liver (presence/absence/unknown)	33/321/105
Cirrhosis (presence/absence/unknown)	323/135/1
Number of curative treatments for HCC before DAA therapy (1/2/3 or more/unknown)	262/88/105/4

Results are expressed as mean ± SD.

AFP, alpha‐fetoprotein; ALT, alanine aminotransferase; APRI, aspartate aminotransferase to platelet count ratio index; AST, aspartate aminotransferase; DAA, direct‐acting antiviral agents.; FIB‐4 index, Fibrosis‐4 index; GGTP, gamma‐glutamyl transpeptidase; HCC, hepatocellular carcinoma

**Table 2:** Patient characteristics with or without HCC recurrence after DAA therapy

	Patients with HCC recurrence (n = 217)	Patients without HCC recurrence (n = 242)	*P*‐value
Age before DAA therapy (y)	74.9 ± 7.7	75.0 ± 7.5	0.9327
Sex (male/female)	138/79	131/111	0.0399
AST level before DAA therapy (IU/L)	61.6 ± 34.7	55.9 ± 27.2	0.0518
ALT level before DAA therapy (IU/L)	50.2 ± 35.8	47.0 ± 28.6	0.2883
GGTP level before DAA therapy (IU/L)	47.4 ± 39.0	50.0 ± 65.8	0.6204
Platelet count before DAA therapy (×10^4^/μL)	10.0 ± 4.8	11.4 ± 5.0	0.0021
AFP level before DAA therapy (ng/mL)	36.3 ± 88.3	21.6 ± 39.0	0.0196
APRI before DAA therapy	2.54 ± 1.85	2.12 ± 1.74	0.0117
FIB‐4 index before DAA therapy	7.70 ± 4.00	6.56 ± 4.23	0.0032
Geno/serotype (1/2/1+2/unknown)	190/26/0/1	217/23/1/1	0.3982
Habitual alcohol intake (presence/absence/unknown)	32/153/32	23/180/39	0.0923
Diabetes mellitus (presence/absence/unknown)	59/150/8	69/165/8	0.7707
Fatty liver (presence/absence/unknown)	16/154/47	17/167/58	0.9555
Cirrhosis (presence/absence/unknown)	169/48/0	154/87/1	0.0011
Number of curative treatments for HCC before DAA therapy (1/2/3 or more/unknown)	98/46/71/2	164/42/34/2	<0.0001

Results are expressed as mean ± SD.

AFP, alpha‐fetoprotein; ALT, alanine aminotransferase; APRI, aspartate aminotransferase to platelet count ratio index; AST, aspartate aminotransferase; DAA, direct‐acting antiviral agents.; FIB‐4 index, Fibrosis‐4 index; GGTP, gamma‐glutamyl transpeptidase; HCC, hepatocellular carcinoma

### 3.2 Incidence of HCC

**Figure 2:**
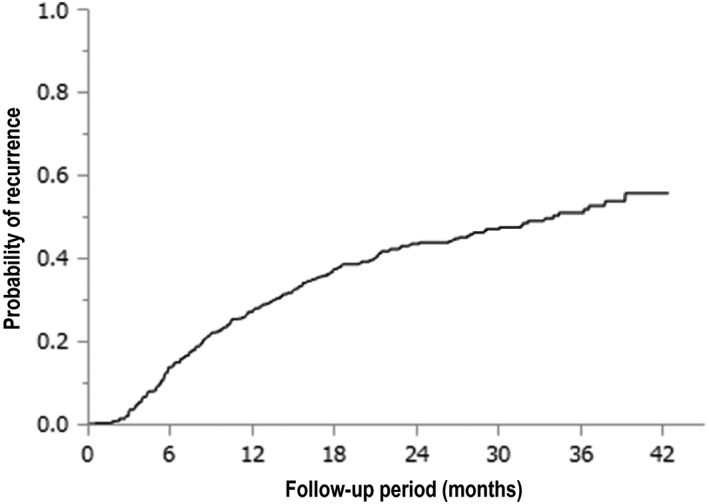
Kaplan‐Meier analysis of the time‐to‐recurrence in the enrolled patients

Of the 459 patients, 217 (47.2%) had developed HCC. Figure 2 shows time‐to‐recurrence in the enrolled patients. The median time‐to‐recurrence was 34.0 months, and the 1‐, 2‐, and 3‐year cumulative HCC recurrence rates were 27.1%, 43.4%, and 50.8%, respectively. Twelve patients (2.6%) died during the observation period. Of these, nine patients had HCC recurrence before they died. When the remaining three patients were excluded from the analysis, the 1‐, 2‐, and 3‐year cumulative HCC recurrence rates were 27.1%, 43.6%, and 51.1%, respectively.

### 3.3 Risk factors associated with HCC recurrence

Table 3 shows the risk factors associated with HCC recurrence after the DAA therapy. Based on the univariate analysis, sex, platelet count, AFP level, APRI, FIB‐4 index, the presence of cirrhosis, and number of curative treatments for HCC before the DAA therapy were identified as factors that were significantly associated with HCC recurrence after the DAA therapy. On the basis of multivariate analysis, the AFP level before the DAA therapy and number of curative treatments for HCC before the DAA therapy were identified as independent factors that were significantly associated with HCC recurrence after the DAA therapy.

**Table 3:** Univariate and multivariate analyses of HCC recurrence after DAA therapy in all patients

	Univariate analysis		Multivariate analysis	
	HR (95% CI)	*P*‐value	HR (95% CI)	*P*‐value
Age before DAA therapy (by every 10 y)	0.986 (0.827‐1.179)	0.8841	0.982 (0.747‐1.291)	0.8992
Sex (female)	1		1	
(male)	1.340 (1.019‐1.774)	0.0357	1.355 (0.958‐1.926)	0.0854
AST level before DAA therapy (by every 10 IU/L)	1.033 (0.991‐1.073)	0.1055	1.066 (0.937‐1.212)	0.3290
ALT level before DAA therapy (by every 10 IU/L)	1.018 (0.976‐1.058)	0.3802	0.996 (0.877‐1.131)	0.9535
GGTP level before DAA therapy (by every 10 IU/L)	0.995 (0.965‐1.018)	0.7610	0.996 (0.950‐1.044)	0.8872
Platelet count before DAA therapy (by every 10^4^/μL)	0.952 (0.922‐0.981)	0.0019	0.995 (0.941‐1.053)	0.8840
AFP level before DAA therapy (by every 10 ng/mL)	1.019 (1.003‐1.031)	0.0046	1.021 (1.006‐1.036)	0.0062
APRI before DAA therapy (by every 1 unit)	1.078 (1.009‐1.147)	0.0195	0.835 (0.561‐1.243)	0.3756
FIB‐4 index before DAA therapy (by every 1 unit)	1.040 (1.011‐1.067)	0.0044	1.054 (0.912‐1.218)	0.4718
Geno/serotype (1)	1		1	
(2)	1.396 (0.904‐2.066)	0.1270	1.128 (0.664‐1.917)	0.6543
Habitual alcohol intake (absence)	1		1	
(presence)	1.384 (0.929‐1.999)	0.1067	1.315 (0.799‐2.165)	0.2808
Diabetes mellitus (absence)	1		1	
(presence)	0.975 (0.716‐1.311)	0.8738	0.939 (0.637‐1.358)	0.7440
Fatty liver (absence)	1		1	
(presence)	1.019 (0.585‐1.652)	0.9407	1.078 (0.569‐1.897)	0.8048
Cirrhosis (absence)	1		1	
(presence)	1.696 (1.240‐2.363)	0.0007	1.332 (0.871‐2.086)	0.1883
Number of curative treatments for HCC before DAA therapy (1)	1		1	
(2)	1.539 (1.074‐2.170)	0.0191	1.608 (1.054‐2.407)	0.0277
(3 or more)	2.561 (1.878‐3.476)	<0.0001	2.610 (1.762‐3.843)	<0.0001

AFP, alpha‐fetoprotein; ALT, alanine aminotransferase; APRI, aspartate aminotransferase to platelet count ratio index; AST, aspartate aminotransferase; CI, confidence interval; DAA, direct‐acting antiviral agents; FIB‐4 index, Fibrosis‐4 index; GGTP, gamma‐glutamyl transpeptidase; HCC, hepatocellular carcinoma; HR, hazards ratio.

### 3.4 AFP level before the DAA therapy

**Figure 3:**
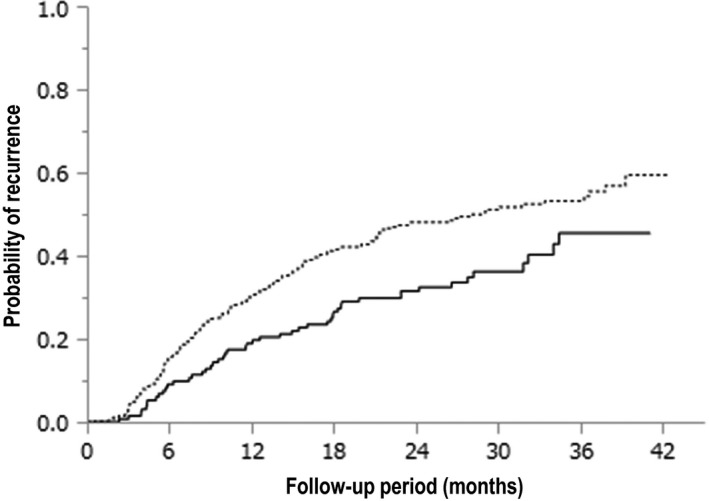
Kaplan‐Meier analysis of the cumulative hepatocellular carcinoma (HCC) recurrence in patients stratified based on serum alpha‐fetoprotein (AFP) levels (*P* = 0.0047). The probability of HCC recurrence in the group with AFP <5.4 ng/mL is indicated by the solid line (n = 132), and that in the group with AFP ≥5.4 ng/mL is indicated by the dotted line (n = 327)

In addition, we determined the cutoff value for the AFP level before the DAA therapy via the receiver operating characteristic (ROC) curve analysis. According to the ROC analysis, AFP = 5.4 ng/ml was identified as the cutoff value. Figure 3 shows the cumulative HCC recurrence stratified with AFP level before the DAA therapy. The 1‐, 2‐, and 3‐year cumulative HCC recurrence rates of the group with AFP level <5.4 ng/mL (solid line; n = 132) were 18.9%, 31.6%, and 45.4%, respectively. The median time‐to‐recurrence in the group with AFP level ≥5.4 ng/mL (dotted line; n = 327) was 29.0 months, and the 1‐, 2‐, and 3‐year cumulative HCC recurrence rates were 30.0%, 48.1%, and 53.2%, respectively. These were found to be statistically significant (*P* = 0.0047).

### 3.5 Number of curative treatments for HCC before the DAA therapy

**Figure 4:**
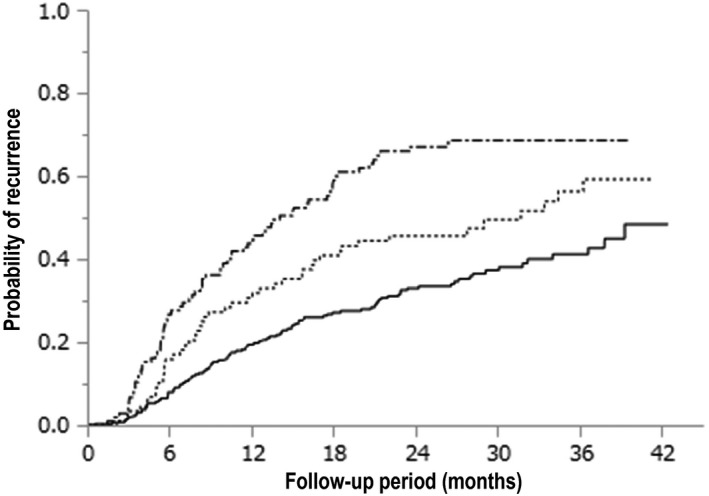
Kaplan‐Meier analysis of the cumulative hepatocellular carcinoma (HCC) recurrence in patients stratified based on the number of curative treatments for HCC before the direct‐acting antiviral agent (DAA) therapy (*P* < 0.0001). The probability of HCC recurrence for once curative treatment for HCC before the DAA therapy is indicated by the solid line (n = 262), that for twice curative treatments for HCC before the DAA therapy is indicated by the dotted line (n = 88), and that for thrice or more curative treatments for HCC before the DAA therapy is indicated by the solid and dotted line (n = 105). The number of curative treatments for HCC before the DAA therapy was unknown in four patients

Figure 4 shows the cumulative HCC recurrence stratified with number of curative treatments for HCC before the DAA therapy. The 1‐, 2‐, and 3‐year cumulative HCC recurrence rates in the group with once curative treatment for HCC before the DAA therapy (the solid line; n = 262) were 19.4%, 33.0%, and 42.0%, respectively. The median time‐to‐recurrence in the group with twice curative treatments for HCC before the DAA therapy (the dotted line; n = 88) was 31.7 months, and the 1‐, 2‐, and 3‐year cumulative HCC recurrence rates were 30.7%, 45.6%, and 56.4%, respectively. The median time‐to‐recurrence in the group with thrice or more curative treatments for HCC before the DAA therapy (the solid and dotted line; n = 105) was 14.0 months, and the 1‐, 2‐, and 3‐year cumulative HCC recurrence rates were 43.8%, 67.1%, and 68.6%, respectively. These were also statistically significant (*P* < 0.0001). Moreover, the number of curative treatments for HCC before the DAA therapy was unknown in four patients.

### 3.6 Correlation between the duration of HCC recurrence and maximum tumor size

**Figure 5:**
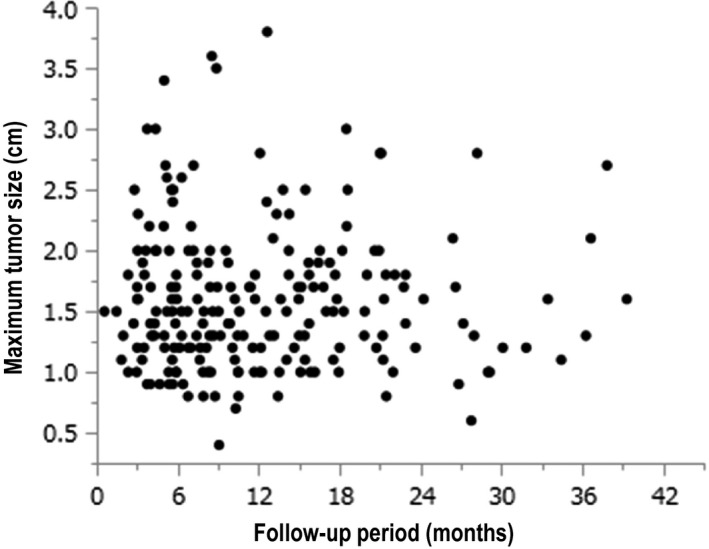
Correlation between the duration of hepatocellular carcinoma (HCC) recurrence and maximum tumor size (r = −0.0130, *P = *0.8517)

Figure 5 shows the lack of correlation between the duration of HCC recurrence and maximum tumor size (r = −0.0130, *P* = 0.8517).

## 4 DISCUSSION

The IFN‐based treatment is often difficult to perform in patients with HCV infection because of the associated adverse events, such as high fever, general fatigue, loss of appetite, and platelet count reduction, especially in the elderly patients and patients with cirrhosis, with a low platelet count. Recently, DAAs have been introduced as an easy and safe antiviral oral therapy for HCV infection. The development of DAAs has rendered the treatment of HCV infection easier in the elderly patients and patients with cirrhosis, with a reduced platelet count, at not only specialized high‐volume centers but also general physician clinics. The eradication of HCV infection has been reported to reduce the risk of HCC.^25^ It is reported that after achieving SVR with DAAs, the risk of developing HCC was the same as that with the IFN‐based therapy.^9^, ^10^, ^11^, ^12^ According to these studies, DAAs do not increase the risk of de novo HCC after achieving an SVR in many patients with the other risk factors of HCC.^10^ Recently, SVR with DAAs has reduced the incidence of HCC in a large prospective study of patients with cirrhosis.^13^

In the present study, the factors associated with HCC recurrence after the DAA therapy were the AFP level and number of curative treatments for HCC before the DAA therapy. In general, the AFP level and frequency of HCC recurrence are suggested to be closely related to malignancy of HCC. An elevated serum AFP level has been reported as a risk factor for HCC in HCV‐infected patients with or without attaining SVR following the IFN therapy.^26^, ^27^, ^28^ An elevated serum AFP level before the DAA therapy has also been recognized as one of the common risk factors for HCC.^26^, ^27^, ^28^ The post‐IFN therapy AFP level was reported as one of the risk factors for developing HCC in patients with old age, advanced liver fibrosis, male sex, glucose metabolism disorders, lipid metabolism disorders, and alcohol intake.^12^ These risk factors have already been reported in patients treated with the IFN‐based therapy.^29^ Especially, the old age, male sex, and advanced liver fibrosis have been reported to be the important risk factors for HCC.^30^, ^31^ However, in our study, age, sex, advanced liver fibrosis, and alcohol intake were not associated with HCC recurrence after the DAA therapy. On the contrary, it suggested that HCC recurrence after the DAA therapy was associated with tumor‐related factors, such as high AFP level and multiple occurrences of HCC before the DAA therapy.

Moreover, in our study, there was no correlation between the duration of HCC recurrence and the maximum tumor size. This suggested the possibility that a relatively large‐sized hepatic tumor will recur relatively early after the DAA therapy. Thus, it is necessary to carefully follow up for recurrence after curative treatment for HCC using image inspection and laboratory data, especially in patients with high AFP level and multiple occurrences of HCC before the DAA therapy.

Our study had several limitations. First, the observation period was short; thus, another study with a longer observation period is needed to confirm our results. Second, the sample size of our study cohort was small; therefore, additional investigations with a higher number of patients and participating institutions are needed.

In conclusion, high AFP level and multiple occurrences of HCC before the DAA therapy indicate a high risk for HCC recurrence after the curative treatment. The DAA‐induced SVR does not always warrant a long HCC‐free status. Thus, follow up after the DAA therapy should include special attention to the abovementioned risk factors.

## CONFLICTS OF INTEREST

Akio Ido has COI as follows: (1) Speaking fees or honoraria; AbbVie GK, Bristol Myers Squibb Co. Ltd., Gilead Sciences Inc, and Eisai Co. Ltd. (2) Research grants; AbbVie GK, Otsuka Pharmaceutical Co. Ltd., MSD KK, Chugai Pharmaceutical Co. Ltd., Eisai Co. Ltd., Astellas Pharmaceutical Inc, and Takeda Pharmaceutical Co. Ltd. The other authors disclose no conflicts.
